# The Mediating Role of Coping Style in the Relationship between Psychological Capital and Burnout among Chinese Nurses

**DOI:** 10.1371/journal.pone.0122128

**Published:** 2015-04-21

**Authors:** Yongqing Ding, Yanjie Yang, Xiuxian Yang, Tiehui Zhang, Xiaohui Qiu, Xin He, Wenbo Wang, Lin Wang, Hong Sui

**Affiliations:** 1 Department of Psychology, the College of Public Health, Harbin Medical University, Harbin, China; 2 The Fifth Affiliated Hospital of Harbin Medical University, Daqing, Heilongjiang Province, China; 3 Department of Surgery, the Fifth Affiliated Hospital of Harbin Medical University, Daqing, Heilongjiang Province, China; 4 Department of Pediatrics, the Fifth Affiliated Hospital of Harbin Medical University, Daqing, Heilongjiang Province, China; 5 Department of Health Statistics, the College of Public Health, Harbin Medical University, Harbin, China; Leibniz Institute for Prevention Research and Epidemiology (BIPS), GERMANY

## Abstract

**Background:**

Burnout is recognized as an occupational hazard, and nursing has a high risk of burnout. This study aims to explore the relationship between psychological capital (PsyCap) and burnout among Chinese nurses and the mediating role of coping style in this relationship.

**Methods:**

A total of 1,496 nurses (effective response rate: 80.11%) from two large general hospitals in Daqing City of China were selected as participants. Data were collected via the Chinese Maslach Burnout Inventory (CMBI), the psychological capital questionnaire (PCQ-24), the Chinese Trait Coping Style Questionnaire (TCSQ) and demographic and caregiver-patient relationship. Hierarchical linear regression analyses were performed to explore the mediating role of positive coping and negative coping, and we used the Bootstrap method to confirm the mediating effect.

**Results:**

Self-efficacy, hope, resilience and optimism of nurses were all negatively related with emotional exhaustion, depersonalization and reduced personal accomplishment among Chinese nurses. Positive coping partially mediated the relationship between hope/optimism and emotional exhaustion and between self-efficacy/optimism and reduced personal accomplishment. Negative coping fully mediated the relationship between self-efficacy and emotional exhaustion, and in the regression model self-efficacy was positively correlated with emotional exhaustion. And negative coping also partially mediated the relationship between hope/optimism and emotional exhaustion and between optimism and depersonalization.

**Conclusion:**

PsyCap had effects on burnout and coping style was a mediator in this relationship among Chinese nurses. Nurses who had a strong sense of self-efficacy adopted more negative coping style, which in turn would lead to higher levels of emotional exhaustion. These findings shed light on the influence of negative coping on burnout, and positive coping was a positive resource for fighting against nurses’ burnout. Hence, in order to avoid negative coping style, improve skill of coping and enhance PsyCap of nurses, active interventions should be developed in the future.

## Introduction

Burnout is a state of mental and physical exhaustion and is defined as a long-term stress response of an individual to prolonged exposure to emotional and interpersonal stressors at work, which is characterized by emotional exhaustion, depersonalization and reduced personal accomplishment [1]. Burnout is recognized as an occupational hazard. It not only leads to lower quality of life and increased risk of secondary diseases such as depressive symptoms, anxiety, musculoskeletal diseases and cardiovascular diseases [2–4] but also relates to worsening job performance, decreased productivity, increased absenteeism and job turnover, and has a negative effect on co-workers [5,6].

As human service professionals, nurses are often required to spend considerable time interacting with patients. And the occupation which nurses engage in is high pressure, high risk, and high labor intensity. So nurses are considered a risk population with high levels of burnout [7,8].Burnout of the nurses not only threatens their own health but causes further dent into the quality care of patients [9].In recent years, Chinese people have paid more and more attention to health and the focus is transforming from disease to health and from sustaining life to quality of life. In addition, the ratio of nurses of China to general population is 1:1750, which is considerably lower than that in developed countries(1:140–1:320) [10].The huge population base, increasing health consciousness and the severe nursing shortage have led to an overload of patients for Chinese nurses. Therefore, nurses have to take on many exhausting and stressful tasks and thus are at high risk of burnout. Exploring the correlative factors of nurses’ burnout is important to reduce burnout, and improve nurses’ health and the quality of health care services in China. There are many influencing factors of burnout. This study focuses on the individual dimension, including psychological capital (PsyCap) and coping style.

Drawn from positive psychology and positive organizational behavior, Luthans and his colleagues proposed the concept of PsyCap, that is, “a positive state of mind exhibited during the growth and development of an individual” [11,12]. PsyCap includes four core parts, i.e., self-efficacy, hope, optimism and resilience. Self-efficacy is defined as the self-confidence inbeing able to perform a task, the ability to face challenges, and the will to succeed. People who are self-confident choose challenging tasks, extend motivation and efforts to successfully accomplish their goals, and persevere when faced with obstacles. Hope refers to the positive motivational state that is based on achieving the intended goals through various pathways. An optimistic individual makes specific attributions for positive events and maintains a positive attitude towards the present and future. Resilience means the capacity to “bounce back” from adversity or even dramatic positive changes. In other words, it is the ability to recover quickly or change and grow from adversities, setbacks and failures. The capacities of all dimensions of PsyCap are measurable, open to development, and can be managed for more effective work performance. Scholars are very concerned about the relationship of PsyCap and burnout, and have done many studies [7–8, 13–17]. Laschinger *et al* identified that increased PsyCap was negatively related to emotional exhaustion, and it was a key variable which may contribute to decreased nurses’ burnout and increased physical and mental well-being [16].

Coping style is another factor which has a significant relationship with burnout [18–22]. Coping is defined as the set of cognitive and behavioral strategies used by an individual to manage the internal and external demands of stressful situations [23].Positive coping and negative coping are diametrically opposed coping style. The individuals who often apply positive coping style do not appraise risks, demands and opportunities as potential threat, harm or loss. Rather, they perceive demanding situations as personal challenges. In this sense, they are not reactive but proactive, as they take constructive actions and create opportunities for growth. These people not only strive for life improvement and build up resources to ensure progress and quality of functioning, but also proactively create better living conditions and higher performance levels. For them, stress is predominantly interpreted as “eustress”[24]. In the work setting, positive coping can generate positive emotions and behaviors which lead to a state of communicative well-being, a professional and personal development, and more personal experiences and resources that increase competence [25].In addition, positive coping can be intended to prevent threatening or harmful situations from arising, and involve accumulation of resources to deal with all conceivable threats [24,26]. Therefore, positive coping is characterized by problem-solving behavior and positive appraisal. In contrast, negative coping is distinguished by applying more emotion-focused coping and palliative coping style. The individuals who apply negative coping style often exhibit distortion of thinking, make negative appraisals and inappropriate self-evaluation (e.g. feeling their inability to tackle problems). They minimize distress through negative ways which focus on negative thoughts (e.g. rumination), attempt to escape stressful situations (e.g. avoidance, denial, and wishful thinking). Studies indicated that positive coping style had a significant positive correlation with PsyCap [27] and high levels of self-efficacy [28]but a negative correlation with burnout, and could negatively predict burnout [19–22]. Negative coping style was positively related to burnout [28]. Emotion-focused coping could predict emotional exhaustion, and reduction in the use of emotion-focused coping might decrease levels of exhaustion [28,29].

Burnout is an individual response to chronic work-related stress [28]. Stress results from the transactional relationship between the person and the environment, and its development is influenced by the processes of appraisal and coping [30,31].Theoretically, PsyCap is a positive state of mind exhibited. Coping is the set of cognitive and behavioral strategies, and burnout is a progressively developed condition arising from the use of the ineffective coping strategies [28,32]. So the positive coping and negative coping styles might play an important role in burnout. This paper aims to explore (1) the relation between PsyCap and burnout; (2) whether the dimensions of coping style mediate the effects of the dimensions of PsyCap on the dimensions of burnout, that is, whether the dimensions of PsyCap affect the dimensions of burnout to some extent via the dimensions of coping style. Specifically, the study hypotheses are as follows:

Hypothesis 1: PsyCap is negatively associated with burnout.

Hypothesis2: Positive coping and negative coping mediate the relations between the dimensions of PsyCap and the dimensions of burnout.

## Methods

### Subjects and procedures

A cross-sectional study was conducted in Daqing of Heilongjiang Province during November and December 2013. Two large general hospitals (>500 beds) were randomly selected from five large general hospitals. In this study 1,867 nurses were recruited and a pool of 1,496 nurses (effective response rate: 80.11%) constituted the potential study sample. All participants signed informed consent forms before completing the measures. The research described in this paper meets the ethical guidelines of the ethics committee of the College of Public Health, Harbin Medical University. And it was approved by the ethics committee of the Fifth Affiliated Hospital of Harbin Medical University and Daqing Oilfield General Hospital. The participants were told about the study goals and the related information that they were engaging in a psychological investigation in which there were no correct or incorrect answers, and individualized data were kept confidential. The questionnaires were completed in a classroom environment.

### Measurement of burnout

The 15-itemChineseMaslach Burnout Inventory (CMBI) revised by Li *et al*.[33]was used to measure the three dimensions of burnout: emotional exhaustion (five items), depersonalization (five items) and reduced personal accomplishment (five items).Items were scored on a seven-point scale ranging from 1 (totally disagree) to 7 (totally agree). All items for reduced personal accomplishment were reversely coded. Studies showed that CMBI was suitable for Chinese cultural background, and had good reliability and validity [34,35].In accordance with the evaluation criterion [35], the cut-off scores for emotional exhaustion, depersonalization and reduced personal accomplishment are greater than 25, 11 and 16, respectively. According to the scores of these three dimensions, the individual burnout is divided into four levels, i.e., no burnout (all of the three dimensions scores are less than cut-off), mild burnout (arbitrary one of the three dimensions scores is greater than or equal to cut-off), moderate burnout (arbitrary two of the three dimensions scores are greater than or equal to cut-off) and severe burnout (all of the three dimensions scores are greater than or equal to cut-off).In this study, the Cronbach’s α value for all 15 items was 0.816. The Cronbach’s α value for emotional exhaustion, depersonalization and reduced personal accomplishment were 0.871, 0.807 and 0.787, respectively.

### Measurement of psychological capital

PsyCap was measured with the psychological capital questionnaire (PCQ-24) which was developed by Luthans et al [36]. The PCQ-24included 24 items and four dimensions: self-efficacy (six items), hope (six items), resilience (six items) and optimism (six items).Items were scored on a six-point scale ranging from 1 (strongly disagree) to 6 (strongly agree). The participants were asked to answer how they felt “right now.” Higher values indicated higher levels of PsyCap.

The Chinese version of the PCQ-24 has been used in Chinese studies and demonstrated satisfactory reliability and validity [37]. In the present study, Cronbach’s alpha coefficient of the total scale was 0.945.Cronbach’s alpha coefficients of self-efficacy, hope, resilience and optimism were 0.906, 0.908, 0.808 and 0.742, respectively.

### Measurement of coping style

The Chinese Trait Coping Style Questionnaire (TCSQ) was used to measure coping style, which included two dimensions: positive coping and negative coping. Each factor was respectively composed of 10 items ranked on a five-point Likert-type scale, ranging from 1 (absolutely no) to 5(absolutely yes).The higher the one-dimensional scores, the more an individual tends to adopt this kind of coping style. The validity and reliability of the TCSQ have been validated [38].In this study, Cronbach’s alpha coefficients of the positive coping and negative coping were 0.790 and 0.776, respectively.

In this study, dependent variables were the three dimensions of burnout: emotional exhaustion, depersonalization and reduced personal accomplishment. Independent variables were the four dimensions of PsyCap: self-efficacy, hope, resilience and optimism. The two dimensions of coping style (positive, negative) were considered to be mediating variables.

### Demographic and caregiver-patient relationship

Demographics included age, gender, education, and marital status. Age was classified as ≤30, 31–40, or ≥41 years. At the time of data gathering, the nurses had worked in hospitals from 6 to 260 months. The education classification was based on the nurses’ educational experiences before they started work and grouped into three different categories: junior college or under, undergraduate, and graduate or above. Marital status was categorized as married (including cohabitation) or single (including those who are unmarried, divorced, separated or widowed). In this study, Effective responses were obtained from 1,496 nurses with 33 males (2.2%) and 1,463 females (97.8%). Caregiver-patient relationship was assessed by the questions, “Have you ever quarreled with patients at work?” and “Have you ever been criticized or threatened by patients or their relatives at work?” with two answers “yes” or “no”.

### Statistical analysis

All analyses were performed using SPSS 19.0 program and all statistical tests were two-sided (α = 0.05).Study variables were compared among age groups and education groups by one-way ANOVA analyses. T-tests were performed to examine the differences in marital status and caregiver-patient relationship status. And Pearson’s correlation coefficient was used to preliminarily inspect the correlations among PsyCap, coping style and burnout.

Baron and Kenny’s analysis technique[39]was used to test the hypothesis concerning the mediating effects of the dimensions of coping style in the relations between the dimensions of PsyCap and the dimensions of burnout. According to Baron and Kenny, the following are the conditions for establishing mediation: (1) the independent variable(Self-efficacy/Hope/Resilience/Optimism) is significantly related to the dependent variable(Emotional exhaustion/Depersonalization/Reduced personal accomplishment); (2) the independent variable is significantly related to the mediator (Positive coping/Negative coping); and (3) the mediator is significantly related to the dependent variable, with the effect of the independent variable on the dependent variableshrinking (partial mediator) or becoming statistically insignificant (full mediator) upon the addition of the mediator to the model.

Before performing the regression analyses, all the continuous variables were centralized in order to avoid multicollinearity [40].

We performed hierarchicallinear regression analysis for each of the burnout dimensions to test the mediating effects. In step 1, the age as the control variable was used because it was assumed to be related to the study variables. In step 2, self-efficacy, hope, resilience and optimism were added. In step 3, positive coping and negative coping were added, respectively. We tested each of the mediator hypotheses according to the preceding approach.

In addition, we conducted bias-corrected bootstrap which was proposed by Preacher and Hayes [41] to test the statistical significance of the mediating effect. Bootstrap method is an increasingly accepted non-parametric and provides a powerful and reasonable method for testing mediation effects [42]. It involves repeatedly sampling from the data set (in this case, 5000 samples were taken), and estimates the indirect effect (or effects, in the case of multiple mediation) in each resampled data set. For each independent variable, if the bias-corrected and accelerated 95% CI (BCa 95% CI) of indirect effect (a*b product) excluded0, it indicated that the mediating role of coping style was statistically significant. Furthermore, if the BCa 95% CI of direct effect of independent variable on dependent variable (c' product) excluded 0, which was statistically significant, it indicated that the mediator variable was a partial mediator. If the BCa 95% CI of c' product included 0, which was statistically insignificant, it indicated that the mediator variable was a full mediator.

## Results

Demographic and caregiver-patient relationship of subjects and distributions of each dimension of burnout in categorical items were shown in Table 1. The mean age of studied population was 32.05 years old (SD = 7.70). Mean emotional exhaustion score differed across age groups (*p*<0.001). Mean emotional exhaustion score (*p*<0.05) and mean reduced personal accomplishment score (*p*<0.01) differed across education groups. 375 nurses (25.1%) ever quarreled with patients and 594 nurses (39.7%) were ever criticized or threatened at work by patients or their relatives. Mean emotional exhaustion score (*p*<0.001) and mean depersonalization score (*p*<0.01) differed across caregiver-patient relationship groups. In this study, the overall prevalence of all degrees of burnout was 74.6%, with 40.0% mild, 27.2% moderate and 7.4% severe burnout.

**Table 1:** Demographic and caregiver-patient relationship of subjects and the distributions of dimensions of burnout in categorical items.

Variable	N(%)	Emotional Exhaustion Mean(SD)	Depersonalization Mean (SD)	Reduced personal accomplishment Mean (SD)
**Age(yr)**		*p* = 0.000	*p* = 0.980	*p* = 0.058
≤30	749(50.1)	21.65(7.97)	9.79(5.60)	15.57(6.03)
31–40	493(33.0)	21.41(8.11)	9.83(5.19)	15.22(6.51)
≥41	254(17.0)	18.62(8.61)	9.87(5.30)	14.46(7.30)
**Education**		*p* = 0.027	*p* = 0.206	*p* = 0.003
Junior college or under	103(6.9)	19.08(8.58)	9.89(6.10)	16.59(5.95)
Undergraduate	635(42.4)	20.99(8.21)	10.09(5.80)	15.66(6.51)
Graduate or above	758(50.7)	21.38(8.11)	9.57(4.96)	14.76(6.38)
**Marital status**		*p* = 0.33	*p* = 0.896	*p* = 0.667
Single	597(39.9)	21.61(8.01)	9.79(5.75)	15.36(5.93)
Married	899(60.1)	20.69(8.31)	9.83(5.19)	15.21(6.74)
**Caregiver-patient relationship**				
Quarrel with patients		*p* = 0.000	*p* = 0.000	*p* = 0.400
Yes	375(25.1)	23.50(7.79)	11.00(5.54)	15.51(5.89)
No	1121(74.9)	20.24(8.17)	9.42(5.32)	15.19(6.60)
Threatened by patients		*p* = 0.000	*p* = 0.002	*p* = 0.853
Yes	594(39.7)	23.45(7.75)	10.35(5.25)	15.30(6.13
No	902(60.3)	19.49(8.11)	9.46(5.50)	15.24(6.20)

### Correlations among PsyCap, coping style and burnout

Results of Pearson correlation analysis were shown in Table 2. Self-efficacy, hope, resilience and optimism had significant negative relationship with emotional exhaustion, depersonalization and reduced personal accomplishment (*p*<0.01). Therefore, the first condition of Baron and Kenny’s technique to test the mediating role of coping style was satisfied in the present study. Self-efficacy, hope, resilience and optimism were significantly related to positive coping and negative coping, but the relationships were different. Self-efficacy, hope, resilience and optimism had a significant positive association with positive coping (*p*<0.01) and a significant negative association with negative coping (*p*<0.01). The second condition of Baron and Kenny’s technique was also met.

**Table 2:** Means, standard deviations (SD) and correlations of continuous variables.

Variables	Mean	SD	1	2	3	4	5	6	7	8
1.Emotional exhaustion	21.06	8.20								
2.Depersonalization	9.81	5.42	0.232**							
3.Reduced personal accomplishment	15.27	6.43	-0.126**	0.109**						
4.Self-efficacy	26.51	5.43	-0.100**	-0.183**	-0.318**					
5.Hope	25.98	5.52	-0.214**	-0.184**	-0.235**	0.751**				
6.Resilience	25.17	4.36	-0.190**	-0.204**	-0.244**	0.683**	0.750**			
7.Optimism	24.55	3.90	-0.184**	-0.252**	-0.222**	0.457**	0.532**	0.590**		
8.Positive coping	35.09	6.94	-0.217**	-0.110**	-0.295**	0.357**	0.388**	0.387**	0.336**	
9.Negative coping	28.98	7.47	0.359**	0.261**	0.077**	-0.150**	-0.195**	-0.212**	-0.220**	-0.106**

**. Correlation is significant at the 0.01 level (2-tailed).

### Association between PsyCap and burnout

Results of the hierarchical multiple regression model of emotional exhaustion were presented in Table 3. Age was significantly negatively related to emotional exhaustion (β = -0.140, *p*<0.001), and explained 1.7% of the variance in it. PsyCap explained 6% of the variance. After controlling for age, self-efficacy (β = 0.265, *p*<0.001) was positively associated with emotional exhaustion, whereas hope (β = -0.352, *p*<0.001) and optimism (β = -0.189, *p*<0.01) were negatively associated with it.

**Table 3:** Hierarchical linear regression analysis results.

	Emotional exhaustion				Depersonalization				Reduced personal accomplishment			
	Step1(β)	Step2(β)	Step3(β)		Step1(β)	Step2(β)	Step3(β)		Step1(β)	Step2(β)	Step3(β)	
Age	-0.140***	-0.117***	-0.113***	-0.097***	0.004	0.024	0.024	0.033	-0.053*	-0.024	-0.020	-0.023
Self-efficacy		0.265***	0.287***	0.247***		-0.064	-0.063	-0.072		-0.356***	-0.333***	-0.357***
Hope		-0.352***	-0.319***	-0.317***		0.004	0.004	0.019		0.078	0.113*	0.079
Resilience		-0.126	-0.088	-0.070		-0.064	-0.063	-0.039		-0.036	-0.003	-0.034
Optimism		-0.189**	-0.145*	-0.097		-0.274***	-0.273***	-0.232***		-0.170**	-0.123*	-0.166**
Positive coping			-0.180***				-0.004				-0.190***	
Negative coping				0.347***				0.155***				0.013
R^2^	0.017	0.077	0.096	0.170	0.000	0.071	0.071	0.114	0.004	0.111	0.145	0.111
ΔR^2^	0.017	0.06	0.019	0.093	0.000	0.071	0.000	0.043	0.004	0.107	0.034	0.000

*p<0.05,

** *p*<0.01,

*** *p*<0.001 (two-tailed).

Results of depersonalization were presented in Table 3. PsyCap explained 7.1% of the variance in depersonalization. Only optimism was significantly negatively associated with it (β = -0.274, *p*<0.001).

Results of reduced personal accomplishment were presented in Table 3. Age was significantly negatively related to reduced personal accomplishment (β = -0.053, *p*<0.05), and explained 0.4% of the variance in it. PsyCap explained 10.7% of the variance. After controlling for age, self-efficacy (β = -0.356,*p*<0.001) and optimism (β = -0.170,*p*<0.01) were negatively associated with it.

### The mediating role of coping style in the relationship between PsyCap and emotional exhaustion

As shown in Table 3, the effects of positive coping (β = -0.180, *p*<0.001)and negative coping(β = 0.347, *p*<0.001)on emotional exhaustion were significantly negative and positive, respectively. In the regression equation, self-efficacy was positively correlated with emotional exhaustion. Hope and optimism were negatively correlated with it.

After adding positive coping in the regression model, the regression coefficients (absolute value of regression coefficient when it is negative) for hope and optimism (from β = -0.352 to β = -0.319, *p*<0.001 and from β = -0.189 to β = -0.145, *p*<0.05, respectively) diminished as shown in the final model. According to the third condition of Baron and Kenny’s technique, positive coping was a partial mediator in the relationship between hope and emotional exhaustion and between optimism and emotional exhaustion.

After adding negative coping in the regression model, the regression coefficients for self-efficacy and hope (from β = 0.265 to β = 0.247, *p*<0.001 and from β = -0.352 to β = -0.317, *p*<0.001, respectively) diminished as shown in the final model. In addition, the change of the regression coefficient for optimism was obvious (from β = -0.189 to β = -0.097, *p*>0.05) and the effect of optimism on emotional exhaustion became statistically insignificant. According to the third condition of Baron and Kenny’s technique, negative coping was a partial mediator in the relationship between self-efficacy and emotional exhaustion and between hope and emotional exhaustion. And negative coping was a full mediator in the relationship between optimism and emotional exhaustion.

As shown in Table 4, the results of bootstrap method showed that the path coefficients of indirect effects of hope and optimism on emotional exhaustion through positive coping were -0.0846 (BCa 95% CI: -0.1200, -0.0539) and-0.1239 (BCa 95% CI: -0.1715,-0.0817), respectively. The path coefficients of direct effect of hope and optimism were -0.1389 (BCa 95% CI: -0.2146, -0.0631) and -0.2629 (BCa 95% CI: -0.3726, -0.1531), respectively. So positive coping was a partial mediator between hope and emotional exhaustion and between optimism and emotional exhaustion.

**Table 4:** Coping style mediating the relation between the dimensions of Psychological capital and the dimensions of burnout.

Dimensions of burnout (Independent Variable)	Dimensions of coping style	Predictor	Pathway	Estimate	SE	95%CI	
						*LL*	*UL*
**Emotional exhaustion**	**Positive coping**	Positive coping on: Hope	a_1_	0.4883	0.030	0.4295	0.5471
		Emotional exhaustion on: Positive coping	b_1_	-0.1732	0.030	-0.2327	-0.1138
		Indirect effects through Positive coping Emotional exhaustion on: Hope	a_1_b_1_	-0.0846	0.017	-0.1200	-0.0539
		Direct effect of Hope on Emotional exhaustion	c_1_’	-0.1389	0.039	-0.2146	-0.0631
		Positive coping on: Optimism	a_2_	0.5979	0.043	0.5129	0.6828
		Emotional exhaustion on: Positive coping	b_2_	-0.2073	0.032	-0.2690	-0.1456
		Indirect effects through Positive coping Emotional exhaustion on: Optimism	a_2_b_2_	-0.1239	0.023	-0.1715	-0.0817
		Direct effect of Optimism on Emotional exhaustion	c_2_’	-0.2629	0.056	-0.3726	-0.1531
	**Negative coping**	Negative coping on: Self-efficacy	a_3_	-0.2065	0.035	-0.2755	-0.1375
		Emotional exhaustion on: Negative coping	b_3_	0.3860	0.027	0.3335	0.4385
		Indirect effects through Negative coping Emotional exhaustion on: Self-efficacy	a_3_b_3_	-0.0797	0.016	-0.1133	-0.0504
		Direct effect of Self-efficacy on Emotional exhaustion	c_3_’	-0.0706	0.037	-0.1428	0.0016
		Negative coping on: Hope	a_4_	-0.2643	0.034	-0.3317	-0.1969
		Emotional exhaustion on: Negative coping	b_4_	0.3566	0.026	0.3047	0.4084
		Indirect effects through Negative coping Emotional exhaustion on: Hope	a_4_b_4_	-0.0943	0.015	-0.1264	-0.0664
		Direct effect of Hope on Emotional exhaustion	c_4_’	-0.1389	0.039	-0.2146	-0.0631
		Negative coping on: Optimism	a_5_	-0.4223	0.048	-0.5171	-0.3275
		Emotional exhaustion on: Negative coping	b_5_	0.3670	0.027	0.3141	0.4200
		Indirect effects through Negative coping Emotional exhaustion on: Optimism	a_5_b_5_	-0.1550	0.022	-0.1985	-0.1141
		Direct effect of Optimism on Emotional exhaustion	c_5_’	-0.2318	0.052	-0.3332	-0.1304
**Depersonalization**	**Negative coping**	Negative coping on: Optimism	a_6_	-0.4223	0.048	-0.5171	-0.3275
		Depersonalization on: Negative coping	b_6_	0.1566	0.018	0.1209	0.1922
		Indirect effects through Negative coping Depersonalization on: Optimism	a_6_b_6_	-0.0661	0.011	-0.0902	-0.0469
		Direct effect of Optimism on Depersonalization	C_6_’	-0.2835	0.035	-0.3517	-0.2152
**Reduced Personal accomplishment**	**Positive coping**	Positive coping on: Self-efficacy	a_7_	0.4552	0.031	0.3947	0.5158
		Reduced Personal accomplishment on: Positive coping	b_7_	-0.1924	0.024	-0.2391	-0.1457
		Indirect effects through Positive coping Reduced Personal accomplishment on: Self-efficacy	a_7_b_7_	-0.0876	0.014	-0.1168	-0.0620
		Direct effect of Self-efficacy on Reduced Personal accomplishment	c_7_’	-0.2878	0.030	-0.3475	-0.2282
		Positive coping on: Optimism	a_8_	0.5979	0.043	0.5129	0.6828
		Reduced Personal accomplishment on: Positive coping	b_8_	-0.2296	0.024	-0.2769	-0.1824
		Indirect effects through Positive coping Reduced Personal accomplishment on: Optimism	a_8_b_8_	-0.1373	0.020	-0.1785	-0.1001
		Direct effect of Optimism on Reduced Personal accomplishment	c_8_’	-0.2284	0.043	-0.3124	-0.1444

As shown in Table 4, the path coefficients of indirect effects of self-efficacy, hope and optimism on emotional exhaustion through negative coping were -0.0797(BCa95% CI: -0.1133, -0.0504), -0.0943 (BCa 95% CI: -0.1264, -0.0664) and-0.1550 (BCa 95% Cl: -0.1985, -0.1141), respectively. The path coefficients of direct effect of self-efficacy, hope and optimism were -0.0706 (BCa 95% CI: -0.1428, 0.0016), -0.1389(BCa 95% CI: -0.2146, -0.0631) and -0.2318(BCa 95% CI: -0.3332, -0.1304), respectively. So negative coping was a partial mediator between hope and emotional exhaustion. Because the direct effect of self-efficacy on emotional exhaustion included 0, which was statistically insignificant, negative coping was a full mediator. Whereas the direct effect of optimism on emotional exhaustion did exclude 0, which was statistically significant, negative coping was a partial mediator.

### The mediating role of coping style in the relationship between PsyCap and depersonalization

As shown in Table 3, the effect of positive coping on depersonalization was statistically insignificant (β = -0.004, *p*>0.05).The effect of negative coping was positive and significant (β = 0.155, *p*<0.001).

After adding negative coping in the regression model, the regression coefficient for optimism (from β = -0.274 to β = -0.232, *p*<0.001) diminished as shown in the final model. According to the third condition of Baron and Kenny’s technique, negative coping was a partial mediator in the relationship between optimism and depersonalization.

As shown in Table 4, the path coefficient of indirect effect of optimism on depersonalization through negative coping was -0.0661 (BCa 95% CI: -0.0902, -0.0469). The path coefficient of direct effect was -0.2835 (BCa 95% CI: -0.3517, -0.2152). So negative coping was a partial mediator between optimism and depersonalization.

### The mediating role of coping style in the relationship between PsyCap and reduced personal accomplishment

As shown in Table 3, the effect of positive coping on reduced personal accomplishment was negative and significant (β = -0.190, *p*<0.001). The effect of negative coping was statistically insignificant (β = 0.013, *p*>0.05).

After adding positive coping in the regression model, the regression coefficients for self-efficacy and optimism (from β = -0.356 to β = -0.333, *p*<0.001 and from β = -0.170 to β = -0.123, *p*<0.05, respectively) diminished as shown in the final model. According to the third condition of Baron and Kenny’s technique, positive coping was a partial mediator in the relationship between self-efficacy and reduced personal accomplishment and between optimism and reduced personal accomplishment.

As shown in Table 4, the path coefficients of indirect effects of self-efficacy and optimism on reduced personal accomplishment through positive coping were -0.0876 (BCa 95% CI: -0.1168, -0.0620) and-0.1373 (BCa 95% CI: -0.1785, -0.1001), respectively. The path coefficients of direct effect of self-efficacy and optimism were -0.2878 (BCa 95% CI: -0.3475, -0.2282) and -0.2284 (BCa 95% CI: -0.3124, -0.1444), respectively. So positive coping was a partial mediator between self-efficacy and reduced personal accomplishment and between optimism and reduced personal accomplishment.

## Discussion

This study investigated the relationship between PsyCap and burnout and the mediating role of coping style in the relationship between the dimensions of PsyCap and the dimensions of burnout among nurses in the general hospitals in Daqing of China. In the present study, the prevalence of burnout of nurses was 74.6%, which was higher than previous studies. Li et al [35] selected 175 nurses (effective response rate: 81.78%) as participants and found that the prevalence of burnout was 69.1%. Ribeiro’s research indicated that the prevalence of burnout of nurses whose scores of at least one dimension are greater than or equal to cut-off was 65.4% [43].

In this research, we found that all dimensions of PsyCap of nurses were negatively related to emotional exhaustion, depersonalization and reduced personal accomplishment. These results validated our hypothesis and the findings were consistent with previous studies [8,11]. Earlier researches also indicated that nurses who possessed higher levels of PsyCap might be less inclined to suffer from burnout, and PsyCap played a critical role in the development of burnout and might effectively decrease the extent of burnout [12,16,17].

PsyCap has not only direct but indirect effect on burnout. In this study, the results of bootstrap method are basically consistent with Baron and Kenny’s technique in the mediating effect of coping style. The bootstrap method is a more advanced method used recently [42]. So the results of bootstrap method were used. Both positive coping and negative coping were mediators in the relationship between PsyCap and burnout. And different coping style mediated the different relationship between dimensions of PsyCap and burnout. Positive coping was found to partially mediate the relationship between hope/optimism and emotional exhaustion and between self-efficacy/optimism and reduced personal accomplishment. Nurses who have higher scores on hope/optimism might have high positive coping, which in turn would lead to lower levels of emotional exhaustion, and nurses who have higher scores on self-efficacy/optimism might have high positive coping, which in turn would lead to lower levels of reduced personal accomplishment. Negative coping partially mediates the relationship between hope/optimism and emotional exhaustion and between optimism and depersonalization. Nurses who have higher scores on hope/optimism might have low negative coping, which in turn would lead to lower levels of emotional exhaustion, and nurses who have higher scores on optimism might have low negative coping, which in turn would lead to lower levels of depersonalization. In addition, negative coping was a full mediator between self-efficacy and emotional exhaustion. And in the regression model, self-efficacy was positively correlated with emotional exhaustion. Nurses who have higher scores on self-efficacy might have high negative coping, which in turn would lead to higher levels of emotional exhaustion. That is, self-efficacy has indirect positive effects on emotional exhaustion through the mediating role of negative coping and negative coping was the sole mediator. Self-efficacy is defined to be individuals’ confidence in their ability to solve a problem or accomplish a task [44], and it has a significant predictive effect on the positive coping style [27]. So the higher the self-efficacy score is, the more the nurses tend to adopt the positive coping style. When encountering knotty problems or facing crises (e.g. saving critical patients or sudden caregiver-patient conflict) and worrying about making errors or trying to avoid negative consequences, the nurses who score high on self-efficacy are also likely to evade or do not take active steps. If the problem is not solved or the external appraisal is negative, nurses might have negative emotion (e.g. sadness, despondency, tedium, irritation, or hopelessness) because of focusing on expectancies for success [45]. When this phenomenon occurs repeatedly, they will gradually become vulnerable to emotional exhaustion. Therefore, if the nurses who have a strong sense of self-efficacy adopt more negative coping style, they in turn may exhibit higher levels of emotional exhaustion. It is thus clear that negative coping plays a very important role in burnout among nurses.

Nowadays, Hospital managers care more about advanced medical technologies. However, for the long-term development of the hospital, the hospital administrators should give special attention to PsyCap and coping skill of nurses. These findings prompt the administrators of hospitals to be aware of the significance of PsyCap and coping style and regard them as an important part of nursing professional development. Especially, nurses should nurture self-confidence, optimism, hope and resilience and develop these attributes at work, and adopt positive coping style so as to correctly handle stressful events.

Our study included a large sample of nurses and the response rate was high. The study therefore allows conclusions towards enhancing nurse’s competency and developing active interventions. But there were some limitations. First, this study is a cross-sectional rather than longitudinal design. Second, because nurses in large general hospitals are more likely to experience burnout, we mostly focused our attention on nurses from these hospitals. Nurses from other types of hospitals will be investigated in our further research.
